# Human Papillomavirus Type Distribution and Correlation with Cyto-Histological Patterns in Women from the South of Italy

**DOI:** 10.1155/2009/198425

**Published:** 2010-01-24

**Authors:** Paola Menegazzi, Luisa Barzon, Giorgio Palù, Elisa Reho, Luigi Tagliaferro

**Affiliations:** ^1^Department of Virology and Molecular Biology, Laboratory “Dr. P. Pignatelli”, Via Martiri d'Otranto, 2, 73100 Lecce, Italy; ^2^Department of Histology, Microbiology, and Medical Biotechnologies, University of Padova, 35121 Padova, Italy

## Abstract

Human papillomavirus (HPV) type-specific distribution was evaluated in genital samples collected from 654 women from the South of Italy undergoing voluntary screening and correlated with cyto-histological abnormalities. HPV DNA was detected in 45.9% of the samples, 41.7% of which had multiple infection and 89.0% had high-risk HPV infection. The prevalence of HPV infection and the rate of multiple infections decreased with age, suggesting natural selection of HPV types with better fitness. In line with other Italian studies, the most common HPV types were HPV-6 and HPV-16, followed by HPV-51, HPV-31, HPV-53, and HPV-66, in women with both normal and abnormal cytology. Cervical intraepithelial lesions grade 2 or 3 were associated with high-risk HPV-16, HPV-18, HPV-31, and HPV-51 infection. These data indicate that prophylactic HPV vaccination is expected to reduce the burden of HPV-related cervical lesions in this population, but also suggest the potential utility of new vaccines with larger type coverage.

## 1. Introduction

With the advent of the HPV vaccine, data on genotype-specific prevalence of HPV infection in sexually active female population would be useful to predict the potential benefits of HPV vaccination and to monitor the impact of vaccination on HPV type replacement. A recent meta-analysis estimated that the worldwide HPV prevalence in the cervix of women with normal cytology is about 10.4% [1]. HPV-16 is the most common HPV type both in women with normal cervical cytology and in those with cervical lesions or cancer, followed by HPV-18 in Europe, Central and South America, HPV-52 and HPV-58 in Asia, and HPV-53 and HPV-52 in Northern America [1, 2]. The pattern of HPV type distribution may vary even among countries and regions, being related to sexual habits and migrations of people, as suggested by detection of HPV types typically found in Asia and Africa and also in southern Europe [2, 3]. 

This cross-sectional study describes HPV prevalence and type distribution in cervical samples from women resident in southern areas of Italy, that is, Apulia region and Naples area, who were referred for opportunistic screening and for gynaecological care for evaluation of suspected HPV-related lesions.

## 2. Material and Methods

### 2.1. Study Population and Specimens Collection

From 2005 to 2008, a total of 654 genital swabs, which were collected from women aged from 17 to 60 years and living in Apulia region (provinces of Lecce, Brindisi, Taranto and Bari) and Naples city in the South of Italy, were analyzed. Women were referred to laboratory “Dr. Pignatelli (Lecce-Italy, departments of Virology & Molecular biology and Cyto-hystopatology) for opportunistic screening and for evaluation of HPV-associated lesions, so this patient population does not represent the general population of women attending public screening programs. The in force Italian privacy law did not allow to collect any data about life-style, sexual partners' number, and so forth. Most samples (87%) were cervical swabs, 3.5% were vulvar swabs, 3% vaginal, and 6.5% other genital swabs. Samples were stored in 1.5 mL saline solution at −20°C until DNA purification.

### 2.2. PCR Amplification of HPV DNA

Total DNA was purified from 150 *μ*L cellular suspension by using an ABI PRISM 6100 System (*Applied Biosystems inc., Monza, Italy*) and eluted in a final volume of 120 *μ*L; 10 *μ*L of each DNA sample were used for HPV PCR analysis. HPV PCR was performed using the SPF_10_ primers (INNOLiPA HPV genotyping, *Innogenetics srl–*Pomezia, Italy), which amplify a 65-bp fragment of the L1 open reading frame and allow detection of at least 28 different HPV types by hybridization on oligonucleotide probes [4, 5], as described in the commercial kit protocol. Appropriate negative and positive controls were included in each session to monitor cross-contamination and efficiency of DNA purification and PCR amplification.

### 2.3. HPV Genotyping by Using the INNO-LiPA HPV Genotyping System

The INNO-LiPA HPV genotyping system can identify the following HPV types: HPV-6, 11, 16, 18, 26, 31, 33, 35, 39, 40, 42, 43, 44, 45, 51, 52, 53, 54, 56, 58, 59, 66, 68, 69/71, 70, 73, 74 and 82. HPV types 16, 18, 26, 31, 33, 35, 39, 45, 51, 52, 53, 56, 58, 59, 66, 68, 73, and 82 were defined high-risk (HR) HPV types, whereas the other types were defined low-risk (LR) HPVs [6]. Recently, HPV classification has been revised and HPV-26, 53, 66, 73, and 82 have been downgraded from the category of probably carcinogenic (Group 2A) to possibly carcinogenic (Group 2B) [7, 8]. HPV genotyping assay was performed as previously described [9]. After drying, the strips were visually interpreted using the reference guide provided, according to the manufacturer's protocol.

### 2.4. Statistical Analysis

The association between HPV infection and clinical variables was assessed through the Chi-square test. A *P* value of less than .05 was considered statistically significant.

## 3. Results

HPV DNA was identified in 300 of 654 samples (45.9%); 175 (58.3%) samples had single infection and 125 (41.7%) had multiple infection. Among HPV-positive samples, 267 (89.0%) had HR-HPV infection (Table 1). HPV-6 and HPV-16 were the most commonly detected types, both in single and multiple infections, followed by HPV-51, HPV-31, HPV-66, and HPV-53. HPV-16 was significantly more frequent in single infection, whereas other HR-HPV types (i.e., HPV-56, HPV-58, and HPV-68) were more frequently identified in multiple infection (Table 1).

 HPV DNA detection rate decreased with age, placing on 56.1% for women under 40 and on 37.7% in older ones (Chi-square trend test, *P* < .0001), as well as the prevalence of multiple HPV infections tended to decrease with age, probably due to selection by immunity or HPV type fitness, as suggested by the different distribution of HPV types in different age groups.

In the 231 women who performed both HPV typing and PAP test, HPV DNA was found in about 50% of negative PAP tests (*n* = 208) and ASC-US (*n* = 11) and in 83.3% of LSIL (*n* = 12; no cases of HSIL were identified). Detection of HR-HPVs was common (80%) both in normal and abnormal Pap smears.

Histological analyses were performed in 86 women who underwent colposcopy and cervical biopsy. HPV DNA was detected in 56.1% of biopsies with negative histology, 60.9% of which had HR-HPVs. The prevalence of HPV-DNA (and HR-HPV DNA) detection progressively increases with the worsening of histological pattern, with 100% CIN-2/3 cases positive for HR-HPVs, including HPV-16, HPV-18, HPV-31, and HPV-51 (Table 2). 

## 4. Discussion

This study on a female population from the South of Italy undergoing HPV testing for opportunistic screening or for evaluation of genital lesions shows a relatively high prevalence of HPV infection, 40% of which involved multiple HPV types. The most frequent HPV types were HPV-6, HPV-16, HPV-51, HPV-31, HPV-53, and HPV-66, with a different distribution in young versus older women, suggesting the existence of a natural selection of HPV types which preserve a better fitness, such as HPV-16 and HPV-31. HR-HPVs were detected in all CIN-2/3 samples, with HPV-16, HPV-18, HPV-31, and HPV-51 as the most frequent types. However, HR-HPV types were detected also in a high rate in women with a negative Pap test or with a negative histology. 

These findings are in agreement with other studies on HPV infection and type distribution in Italian women from different regions and in other European countries. In fact, a high prevalence of HPV infection, mostly caused by HR-HPV types, has been observed also in other Italian regions [10–18]. All studies concordantly demonstrate that HPV-16 is by far the most common type, followed by HPV-66, HPV-31, HPV-51, HPV-52, HPV-53, and HPV-58 (the rating order varies among series), while HPV-18 and HPV-45 are less common [11–18]. At variance with studies on women participating in organized cervical screening programs [10], HPV-6 was frequently detected in our patients, who were often referred for suspected condylomas. 

Similar HPV type distribution has been recently reported from other European countries, such as Denmark [19] and Germany [20], where HPV-16 was shown to be the most common HPV type, followed by HPV-31, HPV-51, HPV-52, HPV-53, and HPV-66. 

In the present study, HR-HPVs, including HPV-16, HPV-18, HPV-31, and HPV-51, were detected in 100% of CIN2/3 lesions. The same distribution of HR-HPV types was identified in high-grade cervical lesions investigated in other Italian regions [12, 18, 21] and European countries [19, 20, 22].

## 5. Conclusion

The results of this paper provide information on HPV type distribution in women from Southern Italy and, by a revision of the recent literature, indicate that the epidemiology of HPV infection in these areas is not different from other Italian regions and from other European countries. Besides HPV-16 and HPV-18, which are targeted by the currently available HPV vaccines, other HR-HPVs, such as HPV-31 and HPV-51, appear to be diffused in the population of sexually active women and to be associated with high-grade cervical lesions. These HPVs should be taken into consideration in the development of enhanced vaccines with larger type coverage.

## Figures and Tables

**Table 1:** HPV type distribution (%) in single and multiple infections.

HPV genotype	Single infections	Multiple infections
	(*n* = 175)	(*n* = 125)
HPV-6	15.4	11.8
HPV-11	0.6	0.7
HPV-16*	14.9	7.2
HPV-18	1.1	2.6
HPV-26	0.0	0.0
HPV-31	8.0	7.6
HPV-33	1.1	1.0
HPV-35	1.1	1.0
HPV-39	3.4	5.6
HPV-40	0.6	1.0
HPV-42	1.1	0.7
HPV-43	0.6	0.0
HPV-44	1.1	2.0
HPV-45	0.0	1.3
HPV-51	9.1	6.9
HPV-52	6.3	6.3
HPV-53	7.4	6.6
HPV-54	6.3	4.6
HPV-56*	0.6	4.6
HPV-58	5.7	5.6
HPV-59	3.4	1.3
HPV-66	7.4	8.2
HPV-68*	0.6	6.3
HPV-69/71	0.0	0.3
HPV-70	2.3	1.0
HPV-73*	0.0	2.3
HPV-74	1.7	3.3
HPV-82	0.0	0.3

*P < .05; single infection versus multiple infection.

**Table 2:** HPV prevalence and type distribution in different histological patterns.

Histological diagnosis	HPV-DNA positive No. (%)	HR-HPV positive No. (%)	HPV types detected in age groups			
			17–30 yr	31–40 yr	41–50 yr	51–60 yr
Negative (*n* = 41)	23 (56.1)	14 (60.9)	HPV-31, 51, 54, 59, 66, 68, 70	HPV-6, 16, 31/54, 39, 51, 52, 53, 54, 56, 58, 66, 74	HPV-6, 31, 39, 51, 52, 53	HPV-6, 18, 54
HPV lesion (*n* = 27)	21 (77.8)	20 (95.2)	HPV-6, 16, 18/39, 31, 39, 40, 52, 56, 66, 68	HPV-6, 16, 18, 31, 35, 39, 42, 44, 51, 52, 53, 58, 68/73	HPV-31, 52, 53	
CIN 1 (*n* = 9)	8 (88.9)	7 (87.5)	HPV-6, 16	HPV-16, 35, 51, 66	HPV-16, 58	
CIN 2-3 (*n* = 9)	9 (100.0)	9 (100.0)	HPV-18, 31/54, 51, 56	HPV-6, 16, 31/54, 51, 53	HPV-16	HPV-16, 31, 51
